# Integrated Patterns of Subjective Job Insecurity: A Multigroup Person-Centered Study

**DOI:** 10.3390/ijerph192013306

**Published:** 2022-10-15

**Authors:** Valerio Ghezzi, Valeria Ciampa, Tahira M. Probst, Laura Petitta, Ivan Marzocchi, Ilaria Olivo, Claudio Barbaranelli

**Affiliations:** 1 Department of Psychology, Sapienza University of Rome, Via dei Marsi, 78, 00185 Rome, Italy; 2 Department of Psychology, Washington State University Vancouver, 14204 NE Salmon Creek Avenue, Vancouver, WA 98686-9600, USA

**Keywords:** cognitive job insecurity, affective job insecurity, person-centered approach, latent profile analysis, employee well-being

## Abstract

Past research attests to the pivotal role of subjective job insecurity (JI) as a major stressor within the workplace. However, most of this research has used a variable-centered approach to evaluate the relative importance of one (or more) JI facets in explaining employee physical and psychological well-being. Relatively few studies have adopted a person-centered approach to investigate how different appraisals of JI co-occur within employees and how these might lead to the emergence of distinct latent profiles of JI, and, moreover, how those profiles might covary with well-being, personal resources, and performance. Using conservation of resources (COR) theory as our overarching theoretical framework and latent profile analysis as our methodological approach, we sought to fill this gap. To evaluate the external validity of our study results, we used employee sample data from two different countries (Italy and the USA) with, respectively, *n* = 743 and *n* = 494 employees. Results suggested the emergence of three profiles (i.e., the “secure”, the “average type”, and the “insecure”) in both country samples. The “secure” group systematically displayed a less vulnerable profile in terms of physical and psychological well-being, self-rated job performance, positive orientation, and self-efficacy beliefs than the “insecure” group, while the “average” type position on the outcomes’ continua was narrower. Theoretically, this supports COR’s notion of loss spirals by suggesting that differing forms of JI appraisals tend to covary within-person. Practical implications in light of labor market trends and the COVID-19 pandemic are discussed.

## 1. Introduction

Our world of work is rapidly changing because of technological advancements, automation, and globalization. Coupled with intermittent economic shocks such as recessions and high inflation, which cause downward pressure on the labor market, as well as a general erosion of social and employment protections [1], it is perhaps not surprising that job insecurity (JI)—a perceived threat to the stability and continuance of one’s job [2]—is a frequent consequence.

Although there is increasing acknowledgement of JI as a subjective phenomenon (e.g., within the fields of economics and public health [3]), decades of research in the field of organizational psychology have mainly relied on variable-centered approaches using these subjective measures of JI, which cannot identify distinct configurations of profiles that may differ considerably from person to person. Variable-centered approaches are based on predetermined combinations of variables of interest and do not adequately examine how these combinations of variables characterize different people [4]. Increasingly, however, researchers are looking toward person-centered approaches to better capture the subjective experience of phenomena, at least for homogeneous subpopulations of people. Therefore, the first aim of our study is to use a person-centered approach (specifically, latent profile analysis (LPA)) to identify homogenous latent subpopulations of employee experiences of job insecurity using multiple forms of appraisals. In so doing, we advance JI literature by integrating multiple approaches and operationalizations that until today have tended to proceed separately in the study of the JI construct.

While LPA can identify consistent patterns of variables that compose quantitatively and qualitatively distinct configurations (e.g., [5]), a subsequent key step is to demonstrate that such profiles consistently generalize across samples, cultures, and economies [6,7]. Despite a variety of researchers calling for cross-cultural research (e.g., [8,9]), we still need to gain knowledge on cross-cultural validity of the constructs and measures to develop more comprehensive theories that span cultural boundaries [8]. In fact, when assessing precarious employment, one of the most difficult challenges is to define a broad notion that can apply to the divergent meanings emerging from different economies and labor markets [10], and when it comes to subjective evaluations of what people mean by JI, more psychological research is needed to generalize constructs and measures across samples and cultures. Despite the prolific amount of research on the consequences of JI [11,12] and antecedents of JI [13,14], within the existent literature the use of a person-centered approach is still deficient, and even minor interest is reserved for the investigation of subjective JI as a complex phenomenon across different socio-economical contexts. Therefore, the second aim of our study is to investigate whether the different emergent profiles of JI appraisals can be replicated across two different country settings (Italy and the United States) with very different labor markets (e.g., Europe’s less flexible dismissal policies coupled with vastly different social safety nets between Europe and the USA) and using data collected across different historical timepoints. To the extent that we replicate our findings across these different contexts and timeframes, this further supports the durability of the observed profiles of job insecurity.

Finally, our third study aim is to engage in a process of construct validation to demonstrate that the emergent profiles meaningfully relate to known covariates of JI [15]. Thus, we will analyze the validity of the identified profiles by testing their differential explanatory role on health- (e.g., job-related health problems), performance- (e.g., self-rated in-role performance), personality- (e.g., positivity and work-related self-efficacy beliefs), and economic-related (e.g., financial inadequacy) employee correlates and criteria. 

In addressing these aims, we first provide a review of the different approaches to measuring JI perceptions, including recent conceptualizations and operationalizations of this construct. We than discuss a theoretical framework that has contributed to explaining JI phenomena and its associations with a variety of correlates and serves as a foundation for our hypotheses on several variables considered in our study. Next, we discuss the extant research evidence identifying the presence of consistent JI patterns from a person-centered perspective, and we finally propose an LPA approach to integrate all typical appraisals of JI for the investigation of their patterns within the employee population. Finally, by identifying consistent patterns of employees’ JI experiences and integrating the knowledge from different perspectives on JI, we highlight opportunities for practitioners and organizations to design more effective interventions on the negative effects of JI.

## 2. Literature Review on Subjective JI and Theoretical Framework

### 2.1. Conceptualizations of the JI Construct

From a psychological perspective, the existing body of research on JI proposes a variety of definitions of the construct that rely on the existence of multidimensional facets of subjective JI [16]. Greenhalgh and Rosenblatt first introduced the definition of one of the most widely used psychological definitions of JI, that distinguishes between its qualitative and quantitative dimensions [17]. Hellgren and colleagues further elaborated on the definition, describing the quantitative dimension as a concern for the job’s long-term continuity, while qualitative JI was defined as a threat to the continuity of crucial job characteristics [18]. Although there is a common agreement in research that they represent distinct facets of insecurity in employment conditions [19], the aspect of its quantitative nature has received the greatest attention [16]. On the other hand, qualitative JI is recognized as a particularly pertinent feature of JI in the evolving workplace of today [13]. In fact, empirical evidence concerned with variable-centered approaches to JI showed that qualitative JI may be somewhat independent of quantitative JI and may perhaps be more salient at the population level, because when measured together, mean levels of qualitative JI are generally higher than levels of quantitative JI [20,21,22,23]. However, recent person-centered studies showed that more at-risk subgroups of employees display more prominent levels of quantitative JI compared to qualitative JI [24,25,26].

Nonetheless, qualitative and quantitative facets of JI are not the only psychological dimensions that emerged from the literature. With regard to the subjective experience of JI, a more recent approach to measure JI emerged from empirical research, which differentiates a cognitive component from an emotional component [11,27]. Specifically, cognitive JI (CJI) is defined as a perceived threat to one’s employment continuity and/or job features related to, for example, working conditions [14], whereas affective JI (AJI) refers to emotional reactions (e.g., anxiety, fear, anger, etc.) that employees feel about the perceived threat to their job loss [28]. However, the literature has largely regarded JI as a purely cognitive phenomenon [28], and most of the research on JI conceptualizations has lacked an affective component [17,29,30]. Despite the predominance of studies on the cognitive component of JI, empirical evidence suggests that the affective component better captures the JI notion [31,32]. Moreover, a more recent meta-analysis on cognitive and affective JI [11] showed that AJI measures had stronger associations with outcomes and correlates than both CJI and mixed JI measures comprising both AIJ and CJI components. Moreover, dimensionality was also demonstrated considering the explained unique variance of AJI in outcomes and correlates above and beyond CJI, showing that it is empirically meaningful to treat these two components as two separate constructs [11]. 

While past decades of the literature on JI have questioned whether JI is mostly a unidimensional [30,33,34] or a multidimensional construct [17,35,36], and recent debates discussed the integration of a qualitative and quantitative component, as well as an integration of affective reactions linked to cognitive aspects of JI, a more recent review of the construct conducted by Shoss has attempted to refine and “clean up” the construct of JI [14]. Accordingly, JI is a subjective future-focused phenomenon that might refer to prospective job loss “as a whole”, as well as to worsening working conditions or loss of future opportunities [14]. Moreover, the author highlighted that the debate on the cognitive approach and the affective approach in measuring JI should still be clarified, considering that affective reactions could mostly reflect the proximal outcome of a cognitive component, rather than representing a distinct facet of JI itself [27]. However, a comprehensive meta-analysis demonstrates the empirical meaningfulness to distinguish between CJI and AJI appraisals, thus making an argument about the integration of these two aspects that should be simultaneously considered to better understand the complexity of the JI phenomenon [11].

Building on these considerations, we thus propose to integrate such dimensions to examine JI as a comprehensive subjective phenomenon, which includes qualitative, quantitative, cognitive, and affective components, that can shape specific individual configurations or patterns of unique individual JI experiences.

### 2.2. Theoretical Framework of JI and Its Correlates

We rely on the conservation of resources (COR) theory as our theoretical framework to investigate well-being, personality, and behavioral correlates of the different JI configurations that emerge in our study [37]. A central assumption of COR is that “individuals strive to obtain, retain, foster, and protect those things they centrally value” [38] (pp. 103–104). Those valuable objects are described as resources, such as individual dispositions or sources of energies, as well as external advantageous conditions, which represent a value per se (“primary resources”) or are fundamental to acquire or to protect other significant resources (“secondary resources”). From the COR theory standpoint, people react negatively to the external environment not only when they lose real resources but also when they perceive a threat of resource loss. According to this theoretical perspective, a stable job, comprising a variety of features (i.e., type of contract, hierarchical role, job tenure, earnings, etc.), represents a set of valuable resources for workers. Thus, employment is per se an important resource that must be protected, not only because it is an essential component of a person’s identity [39] but also because it provides significant psychosocial functions, such as others’ appreciation, interactions, and opportunities for personal development [40]. Consequently, JI may produce several detrimental implications for workers because it represents a threat to the continuity of these resources, in terms of valued job aspects such as career opportunities (qualitative component of JI), or for the job itself (quantitative component of JI). Similarly, JI may produce negative consequences for workers considering the potential emotional reaction to a perceived threat of job security (affective component of JI), as well as the perception of job instability (cognitive component of JI). Several studies have corroborated the assumptions of COR theory, demonstrating that JI is positively associated with a range of indicators of ill-being, such as mental health problems, negative emotions at work, and job dissatisfaction [41,42,43]. For example, previous research has established that AJI was especially associated with burnout, emotional exhaustion, strain, and general and physical health [11]. Moreover, since JI could make financial planning very difficult for individuals and families, JI has been also linked with the experience of financial stress [44,45].

With regard to personality correlates, JI showed negative associations with the way in which individuals perceive themselves (i.e., their personal resources) [46]. Indeed, individuals who lack resources, including those personal aspects that are usually linked to resilience (i.e., self-efficacy and optimism), are more vulnerable to resource loss (i.e., their employment or key features of their job). Moreover, according to the *desperation principle* of COR theory, people engage a defensive mode to protect themselves when their resources are depleted [38]. This mode is frequently aggressive or defensive, and occasionally irrational, such that people are less likely to use functional resources to counteract resource loss (e.g., positivity and self-efficacy beliefs).

Apart from well-being, attitudes, and personality, JI is also associated with several negative behavioral correlates [12,47,48], and, among them, a worse quality of performance is one of the most relevant constructs associated to JI [49], which may have direct detrimental consequences for organizations. Borrowing the principle of *resource loss cycles* conceptualized by the COR theory, in a stress spiral, people and organizations have less resources to counteract resource loss, and these loss spirals also grow because resource loss is more salient than resource gain (*primacy of loss principle*) and because stress arises when resources are lost [37,38]. Accordingly, when a perceived threat of job loss becomes salient, this may lead workers to implement actions to defend their jobs, but these actions are likely to determine performance decrements because, due to the primacy of resource loss over resource gain (*loss principle*), workers have fewer resources available to invest into their work tasks.

Considering the theoretical framework discussed here and the above review of the literature regarding the link between JI and different correlates, in the current study we focus on three main domains of job-related health problems, namely: (1) affective well-being, financial inadequacy, and financial strain as indicators of employee well-being; (2) task self-efficacy, negative emotion self-efficacy, and positivity as personal resources; and (3) self-rated job performance as a behavioral correlate. As proposed by COR theory, we expect that employees reporting the highest levels of different facets of JI (i.e., cognitive, affective, quantitative, and qualitative) would report worse levels of well-being, performance, and individual resources. Conversely, workers reporting average or low levels of job insecurity would be expected to report higher levels of these variables.

### 2.3. Person-Centered Approaches in Studying JI

Latent profile analysis is by definition an exploratory technique that attempts to uncover meaningful groups from observed data [50]. As such, researchers cannot specify a priori hypotheses regarding the specific number or nature of the expected resulting profiles. Indeed, among the variety of studies that applied a person-centered approach to identify configurations of JI profiles within different subgroups, different and contradictory results have been found. 

A first attempt to identify groups of subpopulations with a type of job classified as “good” or “bad” did not confirm the theoretical dual representation of the labor market segmentation [51]. Using more sophisticated methodological techniques (e.g., LPA), researchers have thus identified eight different profiles using a unidimensional measure of JI capturing both affective and cognitive aspects of the construct [52]. Results showed that majority of the employees (75%) belonged to two stable classes across time, two classes with decreasing levels of JI over time, two classes with increasing levels of JI over time, and two classes with a curvilinear trajectory of JI over time [52]. Although they used a longitudinal research design analyzing differences in intraindividual change [53], findings were not lacking some limitations: the attrition rate between adjacent measurement occasions was high and the study sample comprised only highly educated university workers, making the results difficult to generalize to other occupations and samples.

In another recent study using a different methodological approach [54], namely cross-lagged dual process latent Markov model analysis (LMM analysis; [55]) with 3-wave data, five latent states of JI were found. Results highlighted that 3.5% of the respondents were identified as high on the JI measure and even fewer were classified as “depressed” (2.1% of the respondents). Consistent with these previous findings, five different profiles (from relatively secure to relatively insecure) were also identified with a cross-sectional research design using LPA, in which quantitative and qualitative components of JI were measured [24]. However, authors used an overall generic measure for felt qualitative JI, calling for future studies with more conceptual refinement and measures of the construct.

In contrast with previous findings, a three-profile solution of qualitative and quantitative JI (balanced low, balanced high, and a qualitative JI dominant profile) was found using LPA on two different samples of employees in Lithuania [26]. Researchers identified a balanced low, a balanced high, and a qualitative JI dominant profile, with the balanced high and the qualitative JI dominant profiles linked to significantly poorer mental health and job-related well-being.

Using the same LPA approach, another study conducted in Sweden reported four distinct JI profiles: secure or quality-concerned, employment-concerned, insecure, and secure [25]. Although separate measures of qualitative and quantitative dimensions of JI were used, the study sample only comprised full-time workers, excluding temporary contracts that generally suffer from more vulnerable working conditions and restricting the generalizability of the findings. Moreover, the data were collected in Sweden, where the social security system is typically extended in a large scale to people, in comparison with many other southern countries of Europe [56].

With regards to research evidence on LPA using cognitive (CJI) and affective (AJI) components of JI, only one study by Naranjo and colleagues revealed three profiles, identified as the “secure alignment profile”, the “affective JI misalignment profile”, and the “ambivalent JI alignment profile” [57]. Specifically, in both the two country samples considered in their study (i.e., the UK and the USA), only within the “secure alignment profile” employees were reported slightly higher scores of CJI than AJI, and the “ambivalent alignment profile” was characterized by similar scores between CJI and AJI, whereas a larger difference was found within the “affective JI misalignment profile”, in which employees reported considerably higher scores of AJI compared to CJI [57]. Despite these results being consistent with previous findings in terms of the number of emerged profiles, the major limitation was the use of single-item measures, reducing the reliability of JI measurements, and calling for study replications to identify consistent results for a comprehensive knowledge on subjective JI that includes the affective and cognitive component.

Considering all the evidence reviewed above, research findings show a fragmented knowledge on configurations of subjective JI, with inconsistent results on the number and the characteristics of latent profiles identified on the JI phenomenon. However, these inconsistent results may have emerged because of different constructs and measures being used (e.g., unidimensional or single-item scales), not considering a multidimensional and multifaceted perspective on JI, or because different research designs and analytical techniques were applied. 

Acknowledging these considerations and taking into account that the LPA approach is an explorative approach, we propose to identify JI profiles using a multidimensional perspective of subjective JI incorporating qualitative, quantitative, affective, and cognitive perceptions of JI. Such a multidimensional perspective is grounded in strong theoretical and empirical roots (e.g., [11,14,17,18,27]). As such, we could reasonably expect from three to five different profiles, with relatively high score levels of these dimensions on one subgroup of employees, and relatively low score levels of these dimensions on the other extreme opposite subgroup of employees. Consistent with previous findings [24,25,57], we also expect that the subgroup with the lowest scores in all dimensions of JI will report the highest levels of well-being, personal resources, and performance. Conversely, the subgroup with the highest scores in all dimensions of JI will report the lowest levels on these correlates.

### 2.4. The Present Study

The aim of the present study is threefold. First, we aim to identify homogenous latent subpopulations of employees in terms of cognitive, affective, quantitative, and qualitative JI appraisals. In doing so, we adopt a person-centered analytical framework (i.e., LPA) using data from two employee country samples (i.e., Italy and the USA). Previous studies considering fewer JI dimensions provided a very different number of profiles (e.g., [24,26,52,57]), and, to our knowledge, no prior studies considered simultaneously all typical appraisals of JI for the investigation of their patterns within the employee population. However, in line with prior knowledge and prediction based on COR theory, we expect to extract two “opposite” profiles (one with relatively low and one with relatively high levels of JI appraisals) in both country samples.

Second, although no prior studies investigated how different configurations of JI appraisals can be generalized across cultures, we aim to replicate the final LPA solution across country samples. In this regard, we expect that at least the two “opposite” profiles illustrated in the previous paragraph will emerge both for Italian and USA employees. Specifically, we expect that the perceived accumulation of multiple losses (or gains) in valuable resources (e.g., losing the job or part of its benefits and features) is likely to occur similarly across countries [58].

Finally, we aim to characterize the final LPA solution by comparing the different groups of employees both with fundamental correlates, such as self-evaluative personality traits (i.e., positivity) and socio-cognitive dispositions (i.e., self-efficacy beliefs), physical and psychological well-being, and self-rated task performance. In line with COR theory principles, since the salience of multiple losses in valuable resources (which tend to accumulate) are more powerful than resource gain [38], we expect that more vulnerable integrated patterns of JI appraisals will be associated with lower levels of “positive” individual dispositions (i.e., positivity and job-related self-efficacy beliefs), lower physical and psychological well-being, and lower task performance than other employees.

## 3. Materials and Methods

### 3.1. Procedure and Participants

Italian data were collected during the autumn of 2019 with a snowball sampling strategy in the context of a broader study regarding organizational well-being. No incentives were provided to respondents and only employees were recruited. The final questionnaire was administered through a web platform and participation was completely voluntary. In the USA, data were collected in 2015. Participants were recruited to participate in a larger research project on antecedents, moderators, and outcomes of JI on an online human subjects’ crowdsourcing platform (i.e., Amazon Mechanical Turk). Only “high-reputation” participants (i.e., with established track record of providing high-quality data to previous crowd-sourced tasks; see [59]) were recruited. Moreover, to avoid any potential self-selection based on potential participants’ current perceptions of JI and the other focal topics, it was only indicated that participants needed to be currently employed and would be completing a survey about their “work environment”. A small incentive ($2) was offered upon completion of the survey. Due to the anonymity of the data and low risk for participants, investigators of the institutional review boards of both Italy and the USA classified the study as exempt. The study was conducted according to the guidelines of the Declaration of Helsinki.

The Italian sample (henceforth, referred to as the ITA sample) was comprised of 796 employees (M_age_ = 36.07, SD_age_ = 13.04, 51.5% females), while the USA sample consisted of 498 employees (M_age_ = 37.83, SD_age_ = 12.58, 44.7% females). Samples did not differ in terms of age, years of education, tenure in position, or overtime working hours (see Table 1). Relative to the ITA sample, the USA sample comprised a slightly higher proportion of males and, proportionally, more permanent employees. Finally, the average hours of work declared by the employees was slightly higher in the Italian sample.

### 3.2. Measures

#### 3.2.1. Job Insecurity Facets

##### Cognitive Job Insecurity

The nine-item version of the job security index (JSI, nine items; sample item is “*The future of my job is unknown*”) was used to measure cognitive job insecurity, operationalized as “the perceived stability and continuance of one’s job as one knows it” [27] (p. 452). Participants rated a list of adjectives or brief sentences on a three-point scale (yes = 0, ? or I don’t know = 2, no = 3). Four items were negatively worded and five were positively worded. All items were coded or recoded so that higher scores corresponded to higher CJI.

##### Affective Job Insecurity

The nine-item version of the job security survey (JSS, nine items; sample item is “*The stability of my job is stressful*”) was used to measure AJI, operationalized as “evaluative responses one might have to a perceived level of job security” [27] (p. 455). Participants rated a list of adjectives or brief sentences on the same rating scale of the JSI. Additionally in this case, four items were negatively worded and five were positively worded. All items were coded or recoded so that higher scores corresponded to higher AJI.

##### Quantitative and Qualitative Job Insecurity

Six items (three per dimension) from the scale proposed by Hellgren et al. and used in Låstad et al. were administered (sample item for quantitative JI is “*I worry about being able to keep my job*” and the one for qualitative JI is “*I feel worried about my career development in the organization*”) [18,60]. While quantitative job insecurity was operationalized as the “concerns about the future existence of the present job”, qualitative job insecurity refers to “perceived threats of impaired quality in the employment relationship, such as deterioration of working conditions, lack of career opportunities and decreasing salary development” [18] (p. 182). Participants rated the items on a five-point scale (ranging from 1 = strongly disagree to 5 = strongly agree).

#### 3.2.2. Measures for the Examination of LPA Validity

##### Job-Related Health Problems

Job-related health problems were measured using Hanisch’s formative health complaints index [61], a scale comprising 15 health complaints (e.g., wrist problems and eye irritation) experienced by respondents during the past year because of their jobs. Employees responded yes (1) or no (0) to these 15 health complaints. Higher numbers reflect more health problems.

##### Job-Affective Well-Being

Twelve items from the job-affective well-being scale were used to assess the affective reactions to one’s job [62]. Participants were asked to report how often they experienced different emotions associated with their jobs during the previous 30 days. Six items were positive emotions (e.g., excited) while six were negative (e.g., gloomy). Items were coded so that higher scores represented a more positive affective well-being.

##### Financial Inadequacy and Financial Strain

Relying on a prior Delphi study [63], Petitta et al. developed eight items to measure financial inadequacy (namely, the perceived inadequacy to meet current financial obligations; sample item is “*I pay my bills on time*”) and financial strain (i.e., the affective component of financial stress; sample item is “*I worry about having the funds to cover normal monthly expenses*”) [64]. Participants rated the statements on a five-point frequency scale (ranging from 1 = never to 5 = always).

##### Self-Rated Job Performance

Four items from Williams and Anderson were used to measure self-rated job performance in terms of in-role behavior (sample item is “*I adequately complete assigned duties*”) [65]. Respondents rated each item on a five-point frequency scale (ranging from 1 = never to 5 = always).

##### Positivity

The positivity scale (P Scale) was used as a measure of construct positivity, namely the “tendency to view life and experiences with a positive outlook” [66] (p. 701). This scale encompasses eight items (one negatively worded) consisting of simple statements (e.g., “*I look forward to the future with hope and enthusiasm*”), and participants provided their ratings on a five-point scale ranging from 1 (strongly disagree) to 5 (strongly agree).

##### Self-Efficacy Beliefs

Eight items from the work self-efficacy scale were used to measure negative emotional self-efficacy (i.e., the perceived capability to master negative emotions in stressful situations; four items; sample item is “*Avoid to get angry when others are disrespectful to you*”) and task self-efficacy (i.e., the perceived capability to manage job task and work activities; eight items; sample item is “*Work hard on your activities until you reach the expected goals*”) [67]. All items were introduced by the common stem “I am able to…”. Participants were asked to indicate using a seven-point Likert scale (from 1 = not at all to 7 = completely) the score that best represents their degree of confidence in their ability to do each of things described.

### 3.3. Analytic Strategy

As a preliminary phase, we tested alternative measurement models of the four JI scales. Following recommendations provided by Morin et al. [4,68], we compared four alternative factorial structures: a) an oblique confirmatory factor analytic (CFA) model positing four independent albeit correlated factors (i.e., cognitive, affective, quantitative, and qualitative JI factors), with all cross-loadings fixed to zero; b) a bifactor-CFA model with four specific factors for each of the JI “facet” and a general JI factor, all latent covariances fixed to zero; c) a second-order CFA model, positing a higher-order latent dimension explaining the covariances between the four first-order JI facets; d) an oblique exploratory factor analytic model (ESEM); and e) a bifactor-ESEM. Models d) and e) were similar, respectively, to models a) and b), with the exception that all cross-loadings were freely estimated. Since not all models were nested within the CFA model, they were statistically compared by means of the expected cross-validation index (ECVI) [69]. Lower ECVIs are suggestive of better-fitting models. After the best-fitting model was established for each country sample, we evaluated its cross-sample measurement invariance by testing and comparing increasingly restricted models by following Meredith’s steps: configural, metric, scalar, and strict invariance models [70]. Factor scores were stored from the most restrictive factor model and used for the following latent profile analyses. A latent profile analysis (LPA) approach (for a recent overview, see [71]) was applied to derive optimal unobserved configurations of JI facets within individuals. All analyses were performed using Mplus 8.7 [72]. Initially, a series of models positing an increasing number (*k*) of profiles (ranging from *k* = 1 to *k* = 8) were fitted separately to each sample data. While intercepts were allowed to vary across profiles, variances of each dimension were constrained to equality [73]. The optimal number of profiles was established separately per each sample by considering different indices: (1) lower information criteria (i.e., Akaike information criterion, Bayesian information criterion, and sample-size adjusted Bayesian criterion); (2) the Lo–Mendell–Rubin likelihood ratio test (LRT) and the Vuong–Lo–Mendell–Rubin adjusted likelihood ratio test (VLMR): *p* values < 0.01 associated with a specific LPA solution suggests that a solution with *k* − 1 does not fit the data worse; (3) in case of discrepant information between the above criteria, the elbow plot of the information criteria values can be interpreted. Specifically, the point after which the decrease in the values become negligible is suggestive. In addition, we also considered the relative entropy coefficient (>0.70) and the cluster size (at least 5% of each country sample) to ensure an acceptable classification quality of the overall LPA solution [74,75].

Once the optimal number of profiles was determined for each country sample, we conducted a multigroup LPA investigation testing different similarity hypotheses (i.e., configural, structural, dispersion, and distributional multigroup LPA models) [15]. Final individual membership and classification probabilities were derived from the most restrictive acceptable multigroup LPA solution. Internal validity of the final LPA solution was established by evaluating separately per each sample whether (and how much) the final LPA membership explained the scores of the JI facets originally used as input of the LPA models. Furthermore, the association between the final LPA membership and the available sociodemographic variables was evaluated.

Finally, the cross-cultural measurement invariance of the oblique CFA model of the criterion measures was evaluated and factor scores were stored from the most restrictive model and used as dependent variables in a MANOVA model with the LPA membership used as factor in the context of criterion validity analyses of the final LPA solution.

## 4. Results

Since multivariate outliers may lead to the extraction of spurious profiles [76], they were initially removed from each country sample. Specifically, three and four subjects were dropped, respectively, from the Italian and the USA final samples. Next, alternative measurement models of the JI facets were fitted for each country sample. For sake of model parsimony, JSI and JSI items were grouped into three parcels, each based on their corrected item-total correlations by using the same aggregation scheme in both country samples [77]. Since some indicators reflected slightly high positive values of skewness, all models were carried out using robust maximum likelihood (MLR) estimators. The bifactor-ESEM model showed relevant problems of convergence in both samples (i.e., negative variances of specific factors), so it was not considered for further model comparisons. Table 2 shows the fit of the alternative measurement models. As can be noted, ECVI indices suggest that the ESEM model should be preferred in both samples.

Table 3 shows results of measurement invariance of the ESEM models of JI scales. As can be noted, scalar and strict invariance models were partially tenable. Specifically, two intercepts and four error terms were relaxed across groups. Overall, the measurement properties of the latent constructs were very similar across countries. Correlation among ESEM factors ranged from 0.37 to 0.76 in the Italian sample (M = 0.55, SD = 0.14) and from 0.46 to 0.85 in the USA sample (M = 0.66, SD = 1.5). The reliability of ESEM factors (see Table S1 of Supplementary Materials for further details and ESEM factor loadings) was good in both samples (range of McDonald’s ω in the ITA sample 0.90–0.92, M = 0.91, SD = 0.03; range of McDonald’s ω in the ITA sample 0.80–0.92, M = 0.86, SD = 0.05; range of McDonald’s ω in the USA sample 0.84–0.94, M = 0.91, SD = 0.05). Factor scores stored from the most restrictive measurement invariance model (Model 7 of Table 3) were used as input for further latent profile analyses.

**Figure 1:**
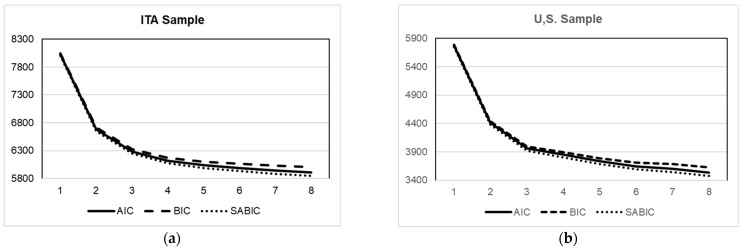
(**a**) Elbow plot of the information criteria of the LPA solutions for the ITA sample. (**b**) Elbow plot of the information criteria of the LPA solutions for the USA sample. Note: *k* number of profiles tested in each LPA solution is reported on the *x*-axis. AIC = Akaike information criterion; BIC = Bayesian information criterion; SABIC = sample-size-adjusted BIC.

Table 4 shows the results of the LPA solutions. While the interpretation of information criteria per se was rather inconclusive (they tend to decrease as the number of profiles increases), LRT and adjusted VLRT tests were significant for *p* < 0.001 in correspondence of the *k* = 3 profile solution, suggesting that a *k* = 3 profile solution may fit better than a *k* = 2 profile solution in both samples. Moreover, the elbow plot of the information criteria (Figure 1) shows that the decrease in AIC, BIC, and SABIC values tends to stabilize after the *k* = 3 profile solution in both samples. Thus, this was retained as the final LPA solution for both the ITA and USA samples. As can be noted, relative entropy coefficients for the *k* = 3 solution (0.879 and 0.965, respectively, for the ITA and the USA samples) were high, ensuring good classification accuracy [74].

Results of multigroup LPA analyses are reported in Table 5. All adjacent models reported a ΔAIC and a ΔBIC > 10, suggesting that more restrictive models fit the data worse than their competitors [78]. The distributional model without cross-cultural profile equality constraints on intercepts and variances was also directly compared with the configural model [15], but also in this case the former model fit the data worse than the latter. Based on the results, we concluded that the three profiles possess a very similar shape across country samples (i.e., configural similarity), but they are different in terms of the mean levels of the clustered variables (i.e., lack of structural similarity), error variances (i.e., lack of dispersion similarity), and the proportion of employees clustered within each configurally similar profile (i.e., lack of distributional similarity) across country samples. For this reason, further validity analyses were conducted separately for each country sample, and the final profile membership was derived from the configural multigroup LPA model, which showed a very high classification accuracy (relative entropy = 0.913).

**Figure 2:**
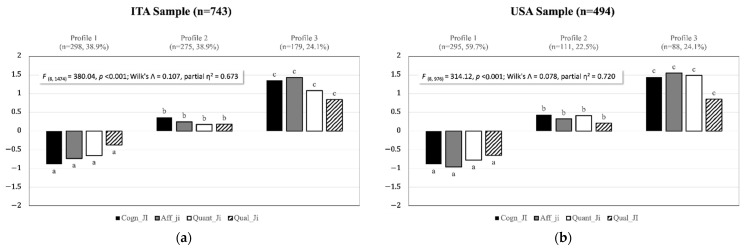
(**a**) Shape and level of the *k* = 3 ITA job insecurity profiles, (**b**) Shape and level of *k* = 3 USA job insecurity profiles. Note: Plotted values represent ESEM factor scores standardized within each country sample. Different subscripts reflect significant differences in MANOVA univariate post hoc tests (Tukey’s HSD). All univariate principal effects were significant for *p* < 0.001.

Figure 2 shows results of the final multigroup LPA configural similarity model. As can be noted, profile membership was strongly discriminative of all JI dimensions in both samples. A first profile (Profile 1, labeled “secure”) represented 38.9% of the ITA and 59.7% of the USA samples. This profile displayed medium-low to low values for all JI dimensions. A second profile (Profile 2, labeled “average type”) represented 38.9% of the ITA and 22.5% of the USA samples. This profile displayed average to slightly high values for all JI dimensions. Finally, the third profile (Profile 3, labeled “insecure”) represented 24.1% of the ITA and 17.8% of the USA samples. This profile displayed medium-high to high values for all JI dimensions. The average latent profile probability for the most likely profile was 0.95, 0.97, and 0.93, respectively, for the “secure”, “average type”, and “insecure” profiles, suggesting a very high degree of separation between the three profiles. The analysis of the standardized residuals that emerged from the country sample profile membership cross-tabulation (χ^2^_(2)_ = 52.78, *p* < 0.001, Cramer’s V = 0.21) revealed a significantly higher proportion of employees classified in the “secure” profile and a lower proportion of employees classified in the “average type” in the USA sample, while no differences were detected for the “insecure” profile.

In the ITA sample, employees’ age classified in the “secure” profile was significantly higher than the other two groups (F_(2657)_ = 19.57, *p* < 0.001, partial η^2^ = 0.06), and the same result was found in the USA sample (F_(2484)_ = 3.50, *p* < 0.001, partial η^2^ = 0.02). Moreover, while employees in the ITA sample with fixed-term employment arrangements were more likely to be classified in the “average type” and “insecure” profiles (χ^2^_(2)_ = 127.61, *p* < 0.001, Cramer’s V = 0.43), only the “insecure” profile was associated with this employment condition in the USA sample (χ^2^_(2)_ = 29.78, *p* < 0.001, Cramer’s V = 0.25).

The final measurement invariance model of criterion measures reflected a full metric, partial scalar, and partial strict equivalence condition (with two intercepts and fiver error terms released across groups, see Tables S2 and S3 of Supplementary Materials for further details). Factor scores were then stored and used in further analyses (latent zero-order correlations and reliability coefficients are reported in Table S4 of Supplementary Materials). All criterion dimensions were internally consistent, with all McDonald’s ω coefficients > 0.78 in the ITA sample and >0.81 in the USA sample.

Table 6 shows the results of criterion validity analyses. With regards to job-related health problems, the profile membership was fully discriminative in the ITA (i.e., the secure profile scored significantly lower than the average type, which, in turn, scored lower than the insecure profile), while in the USA the “insecure” employees did not differ from the “average type” profile (but both displayed significantly higher scores than the secure group). Similar differences were observed for the financial inadequacy criterion. Moreover, LPA profiles were fully discriminative of job-affective well-being in both country samples.

Focusing on financial strain, the LPA solution was fully discriminative in the ITA sample, while the “secure” and the “average type” profiles were not distinguishable in the USA sample (but the “insecure” profile scored significantly higher than both). In both country samples, the “secure” group displayed significantly higher scores on self-rated job performance than “average type” and “insecure” groups (which were not statistically distinguishable in terms of mean differences on the criterion).

While in the ITA sample the “secure” group showed higher scores on positive orientation than the “average type” group, which, in turn, scored significantly higher than the “insecure” group, in the USA sample the “secure” and the “average type” groups showed no mean differences, albeit their scores were significantly higher than those for the “secure” group.

With regards to negative emotional self-efficacy, the “secure” group showed significantly higher scores than the “insecure” group in both country samples (while the “insecure” profile was indistinguishable from the “average type” in relation to this outcome). While same differences in relation to task self-efficacy were found in the USA sample, the “insecure” profile displayed lower scores on this outcome than the other two profiles in the ITA sample. Notably, the “secure” group displayed significant differences from the “insecure” group in all criteria in both country samples.

## 5. Discussion

Drawing upon the COR theory principles [37,38], the present investigation sought to identify latent subpopulations of employees associated with distinct profiles of multiple JI dimensions in two different country samples (i.e., Italy and the USA). In particular, the present study revealed three distinctive latent groups within each sample. We found a low-risk group for JI-related stress (the “secure” profile), comprising 38.9% of ITA employees and 59.7% of the USA sample. A second group (the “average type” profile) classified 38.9% of ITA employees and 22.5% of USA employees, while the most at-risk group for JI-related stress (the “insecure” profile) reported medium-high to high perceptions in all JI dimensions, and it consisted of 24.1% of the ITA sample and 17.8% of the USA participants. Although the three profiles were highly similar across the two samples (high *shape* similarity, see [15]), some relevant differences were observed. For example, the likelihood of being classified in the “secure” group was higher for the USA employees. This result may be explained by a higher proportion of permanent employees in the USA sample, since subjective JI perceptions may be increased by contingent employment statuses [79], and this effect may also translate into less favorable configurations of different JI facets [26]. Moreover, this result may be also partially explained by relevant differences concerned with different labor market conditions and welfare policies across the two countries [56]. A second interesting qualitative difference between the country samples was the “misalignment” between the cognitive and the affective JI facets within the “secure” profile: while in the ITA sample the level of AJI was slightly lower than the CJI, the USA sample revealed the opposite intraindividual pattern. Although these differences were rather weak, this result is consistent with recent findings from Naranjo and colleagues [57] suggesting that cognitive and affective JI perceptions may coalesce with different levels into within-employee configurations. With respect to the combination of quantitative and qualitative JI dimensions within the profiles, both country samples displayed similar results in line with Låstad et al. [25] and De Cuyper et al. [24], where more vulnerable configurations showed systematically higher levels of quantitative JI respect to qualitative JI. This finding was also consistent with the *primacy of loss* principle of the COR theory [37,38]: concerns about the future existence of the present job may represent a disproportionately more salient resource loss than being concerned regarding potential losses in job-related latent and manifest benefits [18]. Interestingly, this finding does not align with evidence provided by studies considering simultaneously quantitative and qualitative JI facets based on variable-centered approaches [20,21,22,23], where qualitative JI scores were higher than quantitative JI ones. This result further corroborates the importance of adopting person-centered approaches as a powerful tool to identify vulnerable subpopulations, which cannot be immediately distinguishable and may display substantial differences from the overall (average) sample characteristics [80].

Despite the differences and peculiarities within and between country samples discussed above, the final *k* = 3 LPA solution displayed three well-separated configurations reflecting three distinct within-employee organizations of JI appraisals both in the ITA and USA samples. With regards to the considered job-related physical and psychological well-being outcomes, results matched our expectations. Specifically, in both samples the “insecure” group reported significantly higher health problems than the “secure” group, as well as for affective well-being. Consistent with the COR theory, employees who perceive to lack in relevant resources (such as job security) are more at risk to develop work-related stress than others and, most importantly, the perceived lack in multiple relevant resources (e.g., the continuance of one’s job or qualitative job-related latent and manifest job-related benefits) may induce employees in a resource loss spiral where they progressively consume their resources to counteract the negative consequences of resource loss on employee physical and psychological health. As documented in previous sections, while most variable-centered studies focused on the relative importance of different JI facets for employee well-being, our results suggested that when cognitive, affective, quantitative, and qualitative appraisals of JI tend to accumulate within the employee, this condition may represent a significant source of vulnerability, resulting in poorer physical and psychological outcomes. Employees with a less favorable configuration of JI dimensions are also more prone to experience both cognitive (*inadequacy*) and affective (*strain*) appraisals of financial insecurity. This finding is consistent with the variable-centered literature concerning the relationship between JI and financial insecurity [81]. Findings of the present study also highlighted that, in both country samples, the “secure” group scored higher than other employees in self-rated job performance, although this difference was weak in both countries. Again, this result is consistent with COR theory, suggesting that more “secure” employees are more likely to invest their resources to prevent and protect themselves from potential resource losses (e.g., the job itself or relevant job-related benefits). Moreover, this finding aligns to recent meta-analytical findings [49], suggesting that employees reporting higher perceptions of job security display higher scores in task performance.

Finally, “secure” and “insecure” employees differed in both countries in terms of positive orientation and self-efficacy beliefs. Specifically, “secure” employees displayed a higher positive orientation and stronger self-efficacy beliefs in mastering negative emotions and tasks within the workplace. Although data of the present study are cross-sectional, these findings may shed light on how self-evaluative dispositions concerned with a positive future orientation and socio-cognitive aspects of individual functioning may represent a basis to understand which employees are more likely to display different integrated patterns of JI appraisals, integrating and completing the variable-centered approach focused on the relationship between stable individual dispositions, self-efficacy beliefs, and JI [82].

### 5.1. Theoretical and Practical Implications

The present contribution expands the current literature in different ways. First, this study integrates the current literature focused on the person-centered approach to JI [83]. Augmenting earlier studies, the current investigation included four well-defined JI facets with a long variable-centered research tradition [13], with the aim to provide preliminary evidence regarding how those qualitatively different dimensions may differently combine within the employee, as well as their characterization in terms of socio-demographic characteristics, well-being, and individual dispositions.

Second, the present contribution represents one of the first attempts to extend the COR theoretical framework [37,38] to the person-centered approach for studying how different JI dimensions configure within the employee. This perspective can be particularly suitable to identify how multiple losses (or gains) in relevant resources may accumulate and whether they can be associated with losses (or gains) in relevant well-being outcomes. Moreover, this approach allows for studying how specific patterns of different JI appraisals (reflecting distinct groups of employees) may differently explain the recovery, adaptation, and deterioration concerned with JI impact on employee overall functioning [83].

Finally, the person-centered approach pursued in the present contribution allows for the identification of differently vulnerable groups of employees in terms of JI appraisals. This may represent a paramount information for managers and policy makers, which may target and promote their interventions (e.g., providing adequate formal and psychological contracts and unambiguous organizational communications, enhancing relevant personal and job resources, and supporting job transitions and reemployment) to specific subpopulations without extending them to the entire employee universe (see [84]).

Such person-centered approaches may be particularly valuable in the aftermath of the coronavirus (COVID-19) pandemic. A recent report on the impact of the coronavirus disease crisis showed that the percentage of temporary contracts in European countries decreased by 17% between spring 2019 and spring 2020, not because the labor market became more stable but because non-renewal of temporary contracts was the first labor market adjustment made by employers [85]. Because of the global pandemic, workers with precarious employment conditions have been particularly exposed to job losses; moreover, the resulting heightened subjective job insecurity in times of financial crisis has never been considered so pivotal as a job stressor (e.g., [3,86,87,88]).

### 5.2. Limitations and Future Directions

Although the present study contributes to the literature, we acknowledge some relevant limitations. First, we used self-reported measures along with a cross-sectional research design. Although we implemented an overall analytical strategy aimed at controlling for multiple sources of measurement error, results may be partially biased because of common method contamination (see [89]). Future studies should integrate the present findings with multiple sources of information from different informants (e.g., employers, supervisors, and colleagues). Moreover, the lack of longitudinal data impedes our ability to evaluate associations between the profiles and the selected outcomes within a prospective time frame. Future studies should replicate the measurement of JI and outcomes over time to evaluate the temporal (in)consistency of the latent subpopulations and their associations with time-lagged outcomes, also developing hypotheses concerned with potential transitions between profiles over time.

Second, although our aim was to replicate our findings across two different contexts to provide further support for the external generalizability of the observed profiles of job insecurity, the Italian and USA samples displayed slightly different results both in terms of variables’ means across configurally similar profiles and their associations with the outcomes. Thus, this suggests that future studies should make more explicit attempts to replicate our findings while focusing on relevant contextual differences. This might include, for example, taking into account potential higher-order differences in cultural values, welfare regimes, and/or job protection practices. Moreover, the two samples from different countries used in the present contribution reflected two Western employee populations (i.e., Italy and the USA). Thus, future research should also aim to replicate the present findings by considering employee populations from other parts of the globe.

## 6. Conclusions

The present contribution sought to identify alternative within-employee configurations of different JI dimensions (i.e., cognitive, affective, quantitative, and qualitative). Using a person-centered theoretical and empirical approach, three latent subpopulations were identified on employee sample data from Italy and the USA (i.e., the “secure”, “average type”, and “insecure” profiles). In line with elaborated predictions consistent with the COR theory, the “secure” profile was less vulnerable than the “insecure” in terms of well-being, task performance, and individual dispositions.

## Figures and Tables

**Table 1:** Sociodemographic Characteristics of the ITA and USA Samples.

*Characteristic*	Italy (N = 796)	USA (N = 498)	Test Statistic	Effect Size
Age (M; SD)	36.07; 13.04	37.83; 12.58	F_(3,1193)_ = 6.14 *	partial η^2^ = 0.01
Gender			χ^2^_(1)_ = 5.32 *	Cramer V = 0.07
Male (%)	48.5	55.3		
Female (%)	51.5	44.7		
Years of education (M; SD)	14.48; 3.14	14.73; 3.48	F_(1,1185)_ = 2.09	partial η^2^ = 0.00
Tenure in position (M; SD)	7.24; 9.06	8.13; 9.17	F_(1,1160)_ = 2.74	partial η^2^ = 0.00
Employment status			χ^2^_(1)_ = 20.13 **	Cramer V = 0.13
Permanent (%)	63.4	75.6		
Fixed term (%)	36.6	24.4		
How many hours do you work in a typical week? (M; SD)	40.63; 9.10	36.27; 10.08	F_(1,1161)_ = 59.96 **	partial η^2^ = 0.05
In a typical week, how many overtime (paid) hours do you work per week? (M; SD)	4.08; 6.19	3.53; 5.74	F_(1968)_ = 2.04	partial η^2^ = 0.00

Note: Fixed-term employment status = fixed-term contracts, apprenticeship training, paid internship, and “do not have a contract”. * *p* < 0.05, ** *p* < 0.001.

**Table 2:** Alternative Measurement Models of Job Insecurity Facets.

	SBχ^2^	*df*	RMSEA (90% C.I.)	CFI	TLI	SRMR	ECVI
ITA Sample (*n* = 743)							
CFA Model	101.369	48	0.039 (0.028–0.049)	0.988	0.983	0.025	0.249
Second-Order CFA Model	142.344	50	0.050 (0.040–0.059)	0.979	0.972	0.043	0.298
ESEM Model	37.792	24	0.028 (0.007–0.044)	0.997	0.991	0.009	0.228
Bifactor-CFA Model	131.697	42	0.054 (0.043–0.064)	0.979	0.978	0.042	0.306
USA Sample (*n* = 494)							
CFA Model	95.987	48	0.045 (0.032–0.058)	0.988	0.983	0.027	0.361
Second-Order CFA Model	150.330	50	0.063 (0.052–0.075)	0.975	0.967	0.056	0.463
ESEM Model	38.063	24	0.034 (0.010–0.054)	0.996	0.990	0.009	0.229
Bifactor-CFA Model	148.942	42	0.072 (0.059–0.084)	0.973	0.958	0.050	0.328

Note: SBχ^2^ = Satorra–Bentler chi-square test statistic; *df* = degrees of freedom; RMSEA = root mean square error of approximation; CFI = comparative fit index; TLI = Tucker–Lewis fit index; SRMR = standardized root mean square residual; ECVI = expected cross-validation index.

**Table 3:** Cross-cultural Measurement Invariance of the ESEM Model of JI Facets.

	SBχ^2^	*df*	RMSEA (90% C.I.)	CFI	TLI	SRMR	Model Comparison	ΔCFI
1. Configural	75.945	48	0.031 (0.016–0.043)	0.997	0.991	0.008	–	–
2. Metric	189.978	80	0.047 (0.038–0.056)	0.988	0.978	0.036	2 vs. 1	0.009
3. Scalar	435.262	88	0.080 (0.072–0.087)	0.958	0.937	0.037	3 vs. 2	0.030
4. Scalar_partial_	250.785	86	0.056 (0.048–0.064)	0.980	0.970	0.033	4 vs. 2	0.008
5. Latent means	315.178	90	0.068 (0.056–0.071)	0.973	0.960	0.058	5 vs. 4	0.007
6. Strict	578.707	102	0.087 (0.080–0.094)	0.943	0.926	0.030	6 vs. 5	0.030
7. Strict_partial_	392.561	98	0.070 (0.062–0.077)	0.965	0.952	0.061	7 vs. 5	0.007

Note: SBχ^2^ = Satorra–Bentler chi-square test statistic; *df* = degrees of freedom; RMSEA = root mean square error of approximation; CFI = comparative fit index; TLI = Tucker–Lewis fit index; SRMR = standardized root mean square residual.

**Table 4:** Fit of the Latent Profile Analyses Conducted for Each Country Sample.

		ITA Sample (*n* = 743)							USA Sample (*n* = 494)						
*k*	*#npar*	LL	AIC	BIC	SABIC	LRT (*p*)	Adj VLRT (*p*)	Entropy	LL	AIC	BIC	SABIC	LRT (*p*)	Adj VLMR (*p*)	Entropy
1	8	−4005	8027	8045	8004	–	–	–	−2879	5774	5792	5751	–	–	–
2	13	−3329	6685	6714	6653	<0.001	<0.001	0.899	−2195	4417	4446	4385	<0.001	<0.001	0.952
3	18	−3124	6284	6325	6245	<0.001	<0.001	0.879	−1961	3959	4000	3920	<0.001	<0.001	0.965
4	23	−3036	6118	6170	6073	0.110	0.116	0.869	−1902	3850	3902	3805	0.594	0.600	0.913
5	28	−2991	6038	6102	5988	0.138	0.142	0.828	−1839	3734	3798	3685	0.029	0.031	0.912
6	33	−2961	5988	6063	5935	0.038	0.041	0.834	−1789	3645	3720	3591	0.090	0.096	0.918
7	38	−2933	5942	6028	5886	0.150	0.158	0.841	−1762	3601	3687	3544	0.692	0.698	0.923
8	43	−2913	5911	6008	5852	0.234	0.244	0.843	−1726	3539	3636	3480	0.220	0.224	0.952

Note: *k* = number of profiles tested in the solution; *#npar* = number of estimated (free) parameters; LL = log-likelihood; AIC = Akaike information criterion; BIC = Bayesian information criterion; SABIC = sample-size-adjusted BIC; LRT (p) = *p* value associated with the Lo–Mendell–Rubin likelihood ratio test statistic; Adj VLMR (*p*) = Vuong–Lo–Mendell–Rubin adjusted likelihood ratio test statistic.

**Table 5:** Fit of the Multigroup Latent Profile Analyses of the *k* = 3 Solution.

Model	#*npar*	LL	AIC	BIC	SABIC	Entropy	Model Comparison	ΔAIC	ΔBIC
1. Configural	37	−5917.78	12,030.27	11,993.27	11,853.52	0.913	–	–	–
2. Structural	25	−5989.68	12,110.93	12,085.93	11,982.31	0.908	2 vs. 1	80.66	92.66
3. Dispersion	21	−6007.46	12,125.44	12,104.44	12,014.03	0.911	3 vs. 2	14.51	18.51
4. Distributional	19	−6030.03	12,160.05	12,141.05	12,057.52	0.911	4 vs. 3	34.61	36.61

Note: *#npar* = number of estimated (free) parameters; LL = log-likelihood; AIC = Akaike information criterion; BIC = Bayesian information criterion; SABIC = sample-size-adjusted BIC.

**Table 6:** Criterion Validity of the *k* = 3 LPA Solution Within Each Country Sample.

Outcome Variable	ITA Sample (*n* = 743)			USA Sample (*n* = 494)		
	F_(df1,df2)_	Partial η^2^	Tukey’s Post Hoc HSD Tests	F_(df1,df2)_	Partial η^2^	Tukey’s Post Hoc HSD Tests
1. Job-Related Health Problems	24.83_(2740)_ ***	0.063	P1 < P2 < P3	8.57_(2490)_ *	0.034	P1 < P2 = P3
2. Job-Affective Well-Being	24.83_(2740)_ ***	0.175	P1 < P2 < P3	28.51_(2490)_ ***	0.104	P1 < P2 < P3
3. Financial Inadequacy	56.18_(2740)_ ***	0.132	P1 < P2 < P3	17.40_(2490)_ ***	0.066	P1 < P2 = P3
4. Financial Strain	76.06_(2740)_ ***	0.171	P1 < P2 < P3	18.45_(2490)_ ***	0.070	P1 = P2 < P3
5. Self-Rated Job Performance	9.45_(2740)_ ***	0.025	P1 > P2 = P3	10.74_(2490)_ ***	0.042	P1 > P2 = P3
6. Positivity	78.85_(2740)_ ***	0.176	P1 > P2 > P3	23.29_(2490)_ ***	0.087	P1 = P2 > P3
7. Negative Emotional Self-Efficacy	7.13_(2740)_ **	0.019	P1 > P2 = P3	21.35_(2490)_ ***	0.080	P1 > P2 = P3
8. Task Self-Efficacy	6.24_(2740)_ ***	0.017	P1 = P2 > P3	16.69_(2490)_ ***	0.064	P1 > P2 = P3

Note: Multivariate effect was F_(16,1466)_ = 16.86, *p* < 0.001; Wilk’s Λ = 0.713, partial η^2^ = 0.155 for the ITA sample, and F_(16,966)_ = 5.95, *p* < 0.001; Wilk’s Λ = 0.829, partial η^2^ = 0.090 for the USA sample. P1 = “secure” profile; P2 = “average type” profile; P3 = “insecure” profile. * *p* < 0.05, ** *p* < 0.01, *** *p* < 0.001.

## Data Availability

Italian data can be provided by the corresponding author upon reasonable request, while USA data can be provided by T.M.P.

## Supplementary Materials

The following supporting information can be downloaded at: https://www.mdpi.com/article/10.3390/ijerph192013306/s1, Table S1: Standardized Factor Loadings and Reliabilities of the ESEM Factors Estimated from the Most Restrictive Measurement Invariance Model; Table S2: Cross-cultural Measurement Invariance of the Correlated CFA Model of the Criterion Validity Measures; Table S3: Standardized Factor Loadings and Reliabilities of the CFA Factors of Criterion Validity Measures Estimated from the Most Restrictive Measurement Invariance Model; Table S4: Latent Zero-Order Correlations and Reliability of the Criterion Validity Measures.
