# Supplementary material for: Integrated Patterns of Subjective Job Insecurity: A Multigroup Person-Centered Study
Source: Int J Environ Res Public Health. 2022 Oct 15;19(20):13306. doi: 10.3390/ijerph192013306 (PMC9602992; doi:10.3390/ijerph192013306)
Supplement: Supplementary file 1 [file ijerph-19-13306-s001.zip › ijerph-1913400-supplementary.pdf]

Supplemental Materials

Integrated Patterns of Subjective Job Insecurity: A Multigroup Person-Centered Study

Valerio Ghezzi[1], Valeria Ciampa[1], Tahira M. Probst[2], Laura Petitta[1],

Ivan Marzocchi[1], Ilaria Olivo[1], Claudio Barbaranelli[1]

[1] Department of Psychology, Sapienza – University of Rome (Italy)

[2] Washington State University, Vancouver, WA (U.S.)

Table S1

Standardized Factor Loadings and Reliabilities of the ESEM Factors Estimated from the Most Restrictive Measurement Invariance Model.

	ESEM Factor 1 Cognitive JI		ESEM Factor 2 Affective JI		ESEM Factor 3 Quantitative JI		ESEM Factor 4 Qualitative JI	
	**ITA**	**U.S.**	**ITA**	**U.S.**	**ITA**	**U.S.**	**ITA**	**U.S.**
**JSI parcel 1**	**.831**	**.830**	.086	.092	-.004	-.005	-.036	-.040
**JSI parcel 2**	**.891**	**.887**	-.029	-.031	.068	.077	.016	.017
**JSI parcel 3**	**.849**	**.898**	.028	.031	-.002	-.003	.028	.032
**JSS parcel 1**	.133	.126	**.764**	**.774**	-.016	-.018	.054	.057
**JSS parcel 2**	-.046	-.049	**.802**	**.902**	.064	.076	-.041	-.048
**JSS parcel 3**	.045	.043	**.826**	**.838**	.001	.001	.028	.029
**Quantitative JI 1**	.060	.062	-.037	-.041	**.759**	**.883**	.027	.031
**Quantitative JI 2**	-.036	-.039	.053	.060	**.752**	**.909**	.006	.007
**Quantitative JI 3**	.066	.060	.030	.029	**.804**	**.831**	-.011	-.011
**Qualitative JI 1**	-.067	-.063	.000	.000	.226	.240	**.550**	**.576**
**Qualitative JI 2**	.018	.017	.026	.026	-.069	-.074	**.812**	**.855**
**Qualitative JI 3**	.025	.023	-.008	-.008	.054	.056	**.824**	**.843**
**Reliability (*McDonald's ω*)**	.916	.935	.870	.915	.836	.932	.800	.838

Note. Factor loadings on the target factor are reported in bold, and they were all significant for $p<.001$.

Table S2

Cross-cultural Measurement Invariance of the Correlated CFA Model of the Criterion Validity Measures.

	$SB\chi^2$	*df*	RMSEA (90% C.I.)	CFI	TLI	SRMR	Model Comparison	ΔCFI
ITA only (n=743)	1437.220	637	.041 (.038 - .044)	.928	.921	.047		
U.S. only (n=494)	1346.248	637	.048 (.044 - .051)	.932	.926	.057		
1. Configural	2776.851	1274	.044 (.041 - .046)	.930	.920	.051	–	–
2. Metric	2859.937	1304	.044 (.042 - .046)	.928	.922	.056	2 vs. 1	.002
3. Scalar	3147.889	1334	.047 (.045 - .049)	.916	.911	.058	3 vs. 2	.012
4. Scalar $_{partial}$	3020.144	1332	.045 (.043 - .047)	.922	.917	.057	4 vs. 2	.006
5. Strict	3402.261	1369	.049 (.047 - .051)	.905	.903	.070	5 vs. 4	.030
6. Strict $_{partial}$	3219.978	1364	.047 (.045 - .049)	.914	.911	.062	6 vs. 4	.008

Note. $SB\chi^2$ = Satorra-Bentler chi-square test statistic; *df* = degrees of freedom; RMSEA = Root Mean Square Error of Approximation; CFI = Comparative fit Index; TLI=Tucker-Lewis fit index; SRMR = Standardized Root Mean Square Residual.

Table S3

Standardized Factor Loadings and Reliabilities of the CFA Factors of Criterion Validity Measures Estimated from the Most Restrictive Measurement Invariance Model.

	Job-Related Health Problems		Job Affective Well-Being		Financial Inadequacy		Financial Strain		Self-Rated Job performance		Positivity		Negative emotional Self-Efficacy		Task Self-Efficacy	
	ITA	U.S.	ITA	U.S.	ITA	U.S.	ITA	U.S.	ITA	U.S.	ITA	U.S.	ITA	U.S.	ITA	U.S.
**JRHP_P1**	.71	.75														
**JRHP_P2**	.76	.80														
**JRHP_P3**	.74	.79														
**JAWS_P1**			.86	.89												
**JAWS_P2**			.86	.89												
**JAWS_P3**			.84	.88												
**FinInad1**					.48	.53										
**FinInad2**					.79	.85										
**FinInad3**					.67	.74										
**FinInad4**					.85	.89										
**FinStrain1**							.81	.82								
**FinStrain3**							.88	.89								
**FinStrain3**							.89	.90								
**FinStrain4**							.86	.87								
**SRJP1**									.76	.82						
**SRJP2**									.78	.89						
**SRJP3**									.86	.90						
**SRJP4**									.72	.87						
**Positivity1**											.57	.80				
**Positivity2**											.80	.85				
**Positivity3**											.54	.61				
**Positivity4**											.74	.79				
**Positivity5**											.83	.87				
**Positivity6**											.47	.53				
**Positivity7**											.72	.77				
**Positivity8**											.66	.83				
**NegEmoSE1**													.78	.79		
**NegEmoSE2**													.81	.82		
**NegEmoSE3**													.66	.67		
**NegEmoSE4**													.68	.69		
**TaskSE1**															.73	.79
**TaskSE2**															.71	.78
**TaskSE3**															.70	.76
**TaskSE4**															.68	.74
**TaskSE5**															.69	.86
**TaskSE6**															.66	.72
**TaskSE7**															.74	.79
**TaskSE8**															.73	.79

Note. Factor loadings were all significant for $p<.001$. For sake of model parsimony, Job-related health problems and job affective well-being items were previously grouped into three parcels based on their corrected item-to-total correlations (Little, 2013).

Table S4

Latent Zero-Order Correlations and Reliability of the Criterion Validity Measures.

	1.	2.	3.	4.	5.	6.	7.	8.
1. Job-Related Health Problems	**.86 (*.89*)**	-.33***	.39***	.29***	-.04	-.20***	-.18**	-.05
2. Job Affective Well-Being	-.27***	**.89 (*.86*)**	-.43***	-.52***	.24***	.63***	.53***	.43***
3. Financial Inadequacy	.21***	-.26***	**.79 (*.85*)**	.85***	-.22***	-.42***	-.31***	-.24***
4. Financial Strain	.25***	-.37***	.77***	**.92 (*.92*)**	-.05	-.49***	-.27***	-.13*
5. Self-Rated Job Performance	-.09	.13**	-.25***	-.08	**.86 (*.92*)**	.31***	.53***	.78***
6. Positivity	-.27***	.50***	-.38***	-.39***	.30***	**.84 (*.92*)**	.54***	.49***
7. Negative Emotional Self-Efficacy	-.16**	.41***	-.08	-.12*	.17***	.31***	**.82 (*.83*)**	.81***
8. Task Self-Efficacy	-.13**	.30***	-.09*	-.04	.51***	.35***	.53***	**.89 (*.92*)**

Note. Correlations below the diagonal pertain to the ITA sample, while those above to the U.S. sample. Reliability coefficients (McDonald's ω) are reported along the diagonal (ITA sample out of brackets, U.S. sample within brackets and italicized). *p<.05, **p<.01, ***p<.001
